# Change in cardiovascular risk factors following early diagnosis of type 2 diabetes: a cohort analysis of a cluster-randomised trial

**DOI:** 10.3399/bjgp14X677833

**Published:** 2014-03-31

**Authors:** James A Black, Stephen J Sharp, Nicholas J Wareham, Annelli Sandbæk, Guy EHM Rutten, Torsten Lauritzen, Kamlesh Khunti, Melanie J Davies, Knut Borch-Johnsen, Simon J Griffin, Rebecca K Simmons

**Affiliations:** MRC Epidemiology Unit, Addenbrooke’s Hospital, Cambridge.; MRC Epidemiology Unit, Addenbrooke’s Hospital, Cambridge.; MRC Epidemiology Unit, Addenbrooke’s Hospital, Cambridge.; Department of Public Health, Section of General Practice, University of Aarhus, Denmark.; Julius Center for Health Sciences and Primary Care, University Medical Center Utrecht, Utrecht, The Netherlands.; Department of Public Health, Section of General Practice, University of Aarhus, Denmark.; Leicester Diabetes Centre, University of Leicester, Leicester.; Leicester Diabetes Centre, University of Leicester, Leicester.; Department of Public Health, Section of General Practice, University of Aarhus and Holbæk Hospital, Holbæk, Denmark.; MRC Epidemiology Unit, Addenbrooke’s Hospital, Cambridge and Primary Care Unit, University of Cambridge, Institute of Public Health, Cambridge.; MRC Epidemiology Unit, Addenbrooke’s Hospital, Cambridge.

**Keywords:** cardiovascular diseases, diabetes mellitus, type 2, prevention and control, primary health care, risk assessment, risk factors, treatment heterogeneity

## Abstract

**Background:**

There is little evidence to inform the targeted treatment of individuals found early in the diabetes disease trajectory.

**Aim:**

To describe cardiovascular disease (CVD) risk profiles and treatment of individual CVD risk factors by modelled CVD risk at diagnosis; changes in treatment, modelled CVD risk, and CVD risk factors in the 5 years following diagnosis; and how these are patterned by socioeconomic status.

**Design and setting:**

Cohort analysis of a cluster-randomised trial (ADDITION-Europe) in general practices in Denmark, England, and the Netherlands.

**Method:**

A total of 2418 individuals with screen-detected diabetes were divided into quartiles of modelled 10-year CVD risk at diagnosis. Changes in treatment, modelled CVD risk, and CVD risk factors were assessed at 5 years.

**Results:**

The largest reductions in risk factors and modelled CVD risk were seen in participants who were in the highest quartile of modelled risk at baseline, suggesting that treatment was offered appropriately. Participants in the lowest quartile of risk at baseline had very similar levels of modelled CVD risk at 5 years and showed the least variation in change in modelled risk. No association was found between socioeconomic status and changes in CVD risk factors, suggesting that treatment was equitable.

**Conclusion:**

Diabetes management requires setting of individualised attainable targets. This analysis provides a reference point for patients, clinicians, and policymakers when considering goals for changes in risk factors early in the course of the disease that account for the diverse cardiometabolic profile present in individuals who are newly diagnosed with type 2 diabetes.

## INTRODUCTION

The promotion of opportunistic screening for diabetes,^1^ coupled with the assessment of diabetes risk in national health checks programmes,^2^ will lead to a greater number of individuals being diagnosed early in the disease trajectory. Among those with established diabetes, the risk of cardiovascular disease (CVD) and mortality can be reduced by intensive treatment of single risk factors, including blood pressure, cholesterol, and glucose.^3^–^6^ Further, a small (*n* = 160) trial of multifactorial treatment found a protective effect at 13 years.^7^ Screen-detected populations have a CVD risk profile that is distinct from that of individuals with clinically diagnosed or established diabetes,^8^,^9^ and evidence to inform the treatment of individuals found earlier in the course of the disease, where CVD risk varies greatly,^8^ is lacking. Results from ADDITION-Europe, a 5-year cluster randomised trial of intensive multifactorial treatment among screen-detected patients, show that it is possible to intensify treatment and reduce levels of many CVD risk factors in this high-risk group.^9^ While the reduction in risk of cardiovascular events associated with the intervention was not statistically significant (hazard ratio = 0.83, 95% confidence interval [CI] = 0.65 to 1.05), there was no increase in modelled CVD risk in the 5 years following diagnosis, despite increasing age and diabetes duration. However, many patients were not prescribed recommended treatments.^8^,^9^ In a screen-detected population that is free of symptoms, primary care teams may be reluctant to prescribe intensive treatment,^10^ and patients may be reluctant to adhere, particularly if they only experience complications related to medications in the short term.^11^ Further, there are examples of inequity in provision of health care for patients with diabetes.^12^,^13^ To inform the development and implementation of treatment policies in this high-risk group, this study aimed to examine baseline CVD risk profiles and treatment of CVD risk factors; change in treatment, modelled CVD risk, and CVD risk factors; and (iii) how these are patterned by socioeconomic status.

## METHOD

This cohort analysis used data from the ADDITION-Europe trial, details of which have been reported previously.^9^ Briefly, ADDITION-Europe is a pragmatic primary care-based trial of intensive multifactorial treatment compared with routine care in those with screen-detected diabetes, in England, Denmark, and the Netherlands. Of 1312 general practices invited to participate, 379 (29%) agreed and 343 (26%) were independently randomised to screening plus routine care of diabetes, or screening followed by intensive multifactorial treatment of CVD risk factors. Screening took place between 2001 and 2006, and out of 3233 individuals found to have undiagnosed prevalent diabetes, 3057 (95%) agreed to take part in the treatment phase of the study.

**How this fits in**

Greater numbers of individuals are being diagnosed early in the diabetes disease trajectory, where there is little evidence to inform treatment. This study shows that the calculation of modelled cardiovascular disease risk is a useful tool for guiding treatment decisions in newly-diagnosed patients with diabetes. Identifying who is at highest risk will help target treatment to those who need it the most and is likely to lead to a reduction in treatment inequity.

Participants underwent a health assessment at baseline, and after a mean of 5.7 years (standard deviation [SD] = 1.3 years) post-diagnosis. Trained staff collected biochemical and anthropometric measurements, according to standard operating procedures.^14^–^16^ Self-report questionnaires were used to collect information on sociodemographic information, lifestyle habits, and medication use. Education was first grouped into tertiles, depending on the age at which participants left full-time education, and then dichotomised into two groups; first versus second and third tertile (low education equals <16 years in the UK and the Netherlands; <21 years in Denmark). Employment status was self-reported.

The characteristics of the interventions to promote intensive treatment in each centre have been described previously and are outlined in Table 1.^14^–^17^ Family doctors, practice nurses, and participants were educated in target-driven management (using medication and promotion of healthy lifestyles) of hyperglycaemia, blood pressure, and cholesterol, based on the stepwise regimen used in the Steno-2 study.^26^

**Table 1.** Treatment protocol for the routine care and intervention groups in ADDITION-Europe

**Setting**	**Routine care**	**Intervention**
Practice (except in Leicester, where patients had access to community-based clinics every 2 months)	Individuals in the routine care group received standard diabetes care according to national guidelines in each country.^18^–^21^ During the course of the study, national guidelines incorporated some elements of the intervention.^22^–^24^	Treatment targets and algorithms were based on trial data.^3^–^6^,^14^ Targets included: keeping HbA1c below 53 mmol/l (7.0%) blood pressure to ≤135/85 mm Hg cholesterol to <5 mmol/l without ischaemic heart disease or <4.5 mmol/l with ischaemic heart disease prescription of aspirin to those treated with antihypertensive medication. The treatment algorithm was amended to include a recommendation to prescribe a statin to all patients with a cholesterol level ≥3.5 mmol/l, following publication of the Heart Protection Study.^25^

### Statistical analysis

Ten-year modelled CVD risk was calculated from the model of the UK Prospective Diabetes Study (UKPDS); version 3 beta),^27^ at baseline and 5-years post-diagnosis. This is a diabetes-specific risk-assessment tool that estimates the absolute risk of fatal or non-fatal CVD within a defined time frame up to 20 years. Participants with complete data on the baseline UKPDS score variables, which are outlined in Box 1, were included in the analyses. The population was divided into quartiles of baseline-modelled CVD risk. Sociodemographic (age, sex, ethnicity, and education), health behaviour (smoking status), health utility (EQ-5D),^28^ and clinical characteristics were summarised by risk quartile and in the cohort as a whole.

**Box 1. The UKPDS cardiovascular disease risk model**

**Background**

A diabetes-specific risk-assessment tool that estimates the absolute risk of fatal or non-fatal CVD within a defined time frame up to 20 years. Participants with complete data on the UKPDS score variables at baseline were assessed.

**Input variables**

Age, sex, ethnicity, smoking status, glycated haemoglobin (HbA1c), systolic blood pressure, total:HDL (high density lipoprotein) cholesterol ratio, atrial fibrillation (AF), previous myocardial infarction or stroke, microalbuminuria (albumin:creatinine ratio ≥2.5 mg/mmol in males, or ≥3.5 mg/mmol in females), macroalbuminuria (albumin:creatinine ratio ≥30 mg/mmol), duration of diagnosed diabetes, and body mass index.

**Notes on use**

There were no data available on AF in ADDITION-Europe participants, so all individuals were coded as zero (no AF). There was a high proportion of missing data for smoking at 5-year follow-up in the Netherlands (29%). Baseline smoking status was used in the calculation of 5-year modelled CVD risk when follow-up values were missing.

Within each modelled CVD risk quartile, the mean absolute change in each CVD risk factor was calculated. To adjust for the differing demographic characteristics of each quartile, centre-specific linear regression models were used to estimate the change in each CVD risk factor within baseline CVD risk quartile, adjusted for age at diagnosis, sex, ethnicity, age of leaving full-time education, randomisation group, and clustering (robust standard errors). Adjusted means for each centre were combined via fixed-effects meta-analysis. The predicted probability of being prescribed any blood pressure-lowering, lipid-lowering, or glucose-lowering medication between diagnosis and 5 years, adjusting for demographic variables (within quartiles of baseline CVD risk), was calculated using a logistic model analogous to the primary analysis model.

Both the overall effect of education and potential interactions between low education and baseline cardiovascular risk were explored using centre-specific regression models as described above. The effect of employment status on change in each CVD risk factor was also examined.

The possibility that observed associations were dependent on the number of quartiles was explored by producing scatter plots of change in each risk factor by baseline modelled CVD risk. The study also explored whether the relationship between baseline risk quartile and risk factor change differed by trial group. Results were similar and trial groups were combined into a single cohort with adjustment for trial group. A multilevel logistic model (practices within centres) was used to explore sociodemographic information that predicted loss to follow-up. Regression to the mean within quartiles was explored by plotting baseline values against change scores.^29^ Data were analysed using Stata (version 12.1).

## RESULTS

### Participant characteristics

At 5 years, 196 people had died, 48 had independently adjudicated cardiovascular-related deaths before 5-year follow-up, and 443 individuals did not have complete data to calculate the UKPDS risk score at baseline. Baseline sociodemographic characteristics were similar between individuals who were included in the analysis (*n* = 2418) and those who were excluded because of missing clinical data at baseline or follow-up (*n* = 443), except for sex (females were more likely to have missing data than males [odds ratio = 1.3; 95% CI = 1.04 to 1.6]). Modelled risk at baseline was missing for 15.5% of the population, while missing data at 5 years ranged from 29% for systolic blood pressure to 37% for albumin:creatinine ratio (ACR).

### Modelled 10-year CVD risk

Compared to the highest-risk quartile, people in the lowest-risk quartile were more likely to be female (67% versus 19%) and younger (56 years, SD = 7.2 years versus 63 years, SD = 5.5 years) and to be more highly educated (54% versus 33%). Individuals at low risk were also more likely to be non-smokers (86% versus 62%), to be free of CVD, and to have more favourable clinical characteristics (Table 2). The proportion of the population prescribed cardioprotective medication (lipid-, glucose- or blood pressure-lowering medication) at baseline was similar across the four quartiles (Table 2).

**Table 2.** Participant characteristics at diagnosis by modelled CVD risk quartile

**Characteristic**	***n* (%)^a^**	**10-year modelled CVD risk by quartile and overall at diagnosis**				

		**<25th centile (Q1)**	**25th to 49th centile (Q2)**	**50th to 75th centile (Q3)**	**>75th centile (Q4)**	**Combined**
**Self-reported**						
% Female	2418 (84.5)	67.4	47.0	32.7	18.7	41.5
Mean (SD) age at diagnosis, years	2418 (84.5)	56.4 (7.2)	59.9 (6.6)	61.5 (6.1)	62.9 (5.5)	60.2 (6.8)
White ethnicity, %	2418 (84.5)	90.6	94.0	94.7	97.5	94.2
Low education, %	1853 (64.8)	39.2	39.7	46.8	52.7	44.5
Current smoker, %	2389	13.6	23.0	30.0	37.8	26.0
Median (IQR) units of alcohol per week	2141 (74.8)	4 (1 to 10)	4 (1 to 13)	5 (1 to 14)	5 (1 to 14)	4 (1 to 12)
Mean (SD) EQ-5D score	2312 (80.8)	0.82 (0.22)	0.84 (0.20)	0.85 (0.20)	0.82 (0.22)	0.83 (0.21)
% Prescribed any glucose-lowering drug^b^	2378 (83.1)	0.7	0.3	0.8	0.5	0.6
% Prescribed any lipid-lowering drug	2378 (83.1)	15.1	16.1	14.4	19.9	16.4
% History of myocardial infarction	2292 (80.1)	0.2	1.6	4.5	17.8	6.0
% History of stroke	2254 (78.8)	0.2	0.7	1.5	6.1	2.1

**Clinical**						
Mean (SD) BMI, kg/m^2^	2418 (84.5)	31.0 (5.7)	31.5 (5.6)	32.0 (5.6)	32.0 (5.1)	31.6 (5.5)
Median (p25 to p75) HbA1c, %	2418 (84.5)	6.2 (5.9 to 6.7)	6.5 (6.1 to 7.0)	6.7 (6.2 to 7.6)	7.2 (6.6 to 9.2)	6.6 (6.1 to 7.4)
Mean (SD) systolic BP, mmHg	2418 (84.5)	137 (17)	146 (18)	153 (20)	161 (24)	149 (22)
Mean (SD) total:HDL cholesterol ratio	2418 (84.5)	3.8 (1.1)	4.4 (1.2)	4.9 (1.3)	5.7 (1.6)	4.7 (1.5)
Median (p25 to p75) triglycerides, mmol/l	2417 (84.5)	1.4 (1.0 to 1.9)	1.5 (1.1 to 2.1)	1.7 (1.3 to 2.4)	2.1 (1.5 to 3.0)	1.6 (1.2 to 2.4)
Median albumin creatinine ratio (p25 to p75), mg/mmol	2259 (79.0)	0.7 (0.3 to 1.4)	0.8 (0.4 to 1.5)	0.9 (0.4 to 2.0)	1.4 (0.6 to 3.5)	0.9 (0.4 to 2.0)
Minimum – maximum 10-year modelled CVD risk at baseline	2418 (84.5)	4.0–17.4	17.4–24.9	24.9–34.9	34.9–92.7	—
Experienced CVD event during follow-up, %	2418 (84.5)	2.1	4.3	6.8	11.3	6.1

BMI = body mass index. BP = blood pressure. CVD = cardiovascular disease. HbA1c = glycated haemoglobin. HDL = high-density lipoprotein. IQR = interquartile range. SD = standard deviation. UKPDS = UK Prospective Diabetes Study.

a Number with variable and complete baseline UKPDS risk score (% included in the study).

b A few participants were offered glucose-lowering medication before confirmatory diabetes diagnosis, owing to high blood glucose values at screening.

**Figure 1.**
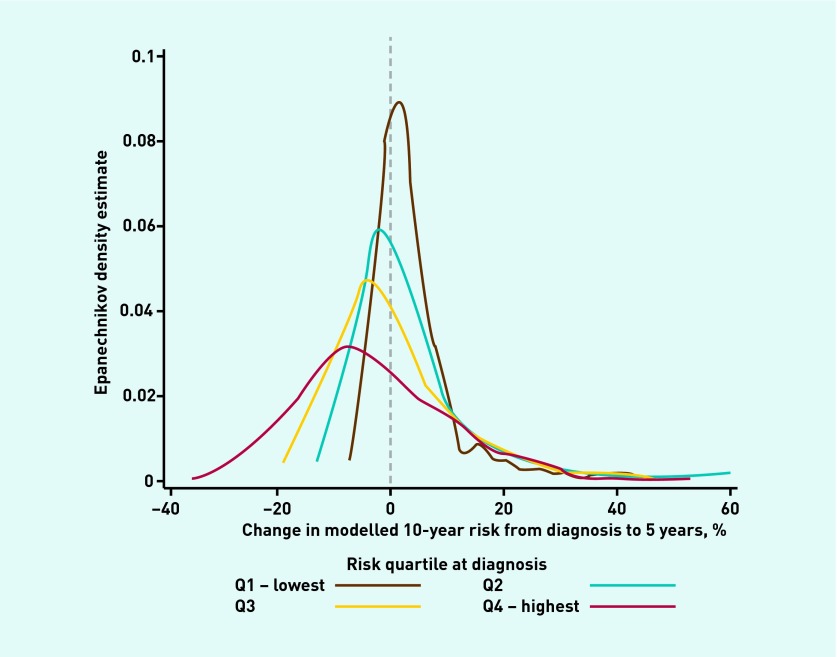
***Distribution of change in modelled CVD risk from diagnosis to 5 years, by quartile of modelled CVD risk at diagnosis.***

Figure 1 shows the distribution of change in modelled CVD risk from baseline to 5-year follow-up. Participants in the highest quartile of CVD risk at baseline showed the largest reduction in CVD risk, and the largest variation in change. Participants in the lowest quartile of modelled risk at baseline had very similar levels of CVD risk at 5-year follow-up and showed the least variation in risk change.

### Body mass index

**Figure 2.**
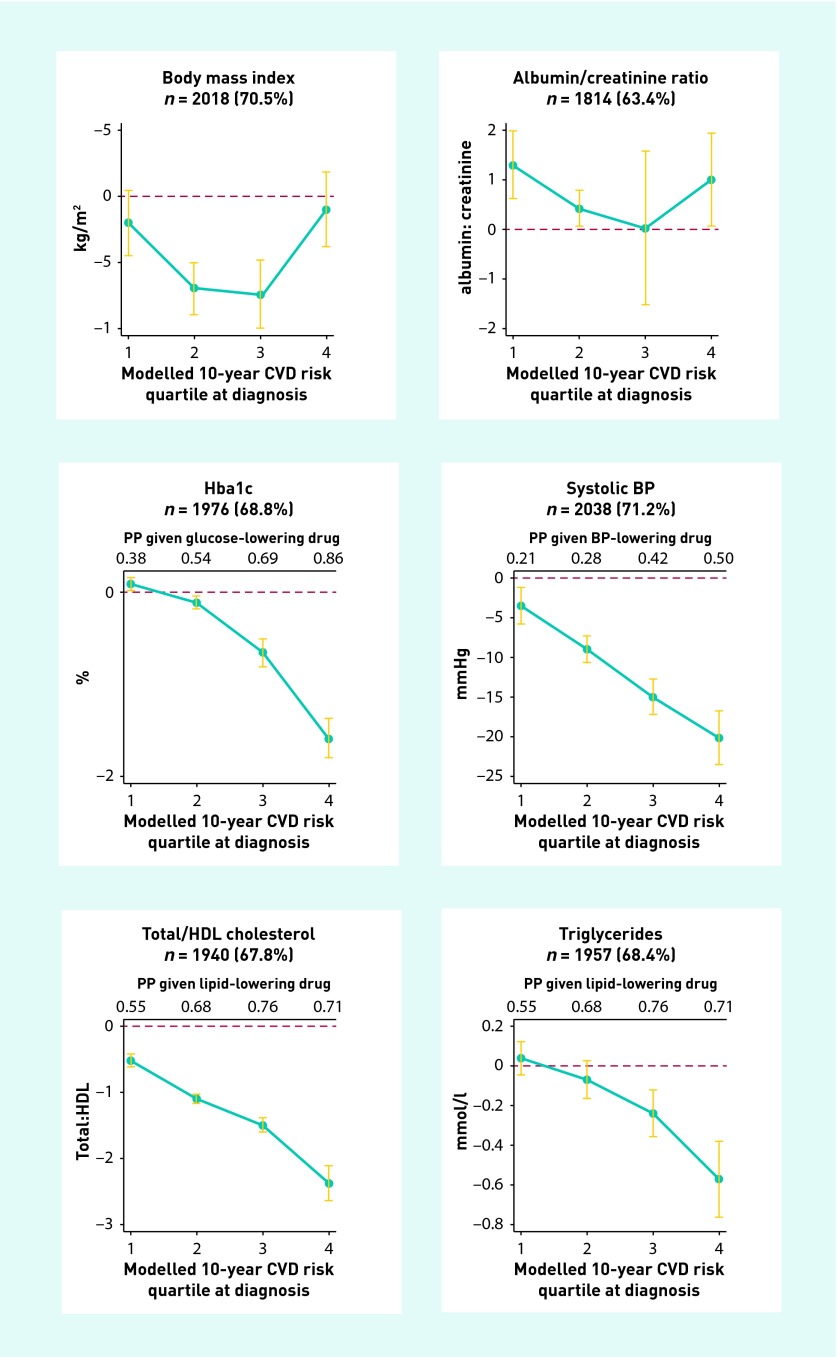
***Absolute change from diagnosis to 5 years (with 95% CI), by modelled CVD risk quartile at diagnosis, adjusted for age, ethnicity, age of leaving full-time education, sex, randomisation group, and practice and centre clustering. Q1, 0–24th centile; Q4, 75–100th centile. BP = blood pressure. PP = predicted probability of being prescribed the medication at 5 years (if not on the drug at baseline), in an adjusted model analogous to the primary analysis.***

Adjusted reductions in body mass index (BMI) were largest among participants in the second (Q2) and third quartile (Q3) for modelled CVD risk (Q2: −0.7 kg/m^2^; 95% CI = −0.9 kg/m^2^ to −0.5 kg/m^2^; Q3: −0.7 kg/m^2^; 95% CI = −0.1 kg/m^2^ to −0.5 kg/m^2^; Figure 2). No significant reductions were observed in Q1 and Q4 (Table 3).

**Table 3.** Adjusted and unadjusted change between diagnosis and 5 years in CVD risk factors, by modelled CVD risk quartile at diagnosis

**Characteristic**	**Baseline modelled CVD risk**				**Combined**

	**<25th centile (Q1)**	**25th to 49th centile (Q2)**	**50th to 75th centile (Q3)**	**>75th centile (Q4)**	
**Unadjusted change**					
BMI, kg/m^2^ (SD)	−0.3 (2.42)	−0.6 (2.40)	−0.84 (2.62)	−0.4 (2.74)	−0.53 (2.56)
Mean (SD) systolic BP, mmHg	−6.12 (18.4)	−9.61 (21.33)	−15.69 (21.51)	−19.92 (25.35)	−12.76 (22.38)
Mean HbA1c, % (SD)	0.17 (0.97)	−0.1 (1.13)	−0.42 (1.54)	−1.19 (1.91)	−0.38 (1.52)
Mean (SD) total cholesterol:HDL ratio	−0.67 (1.06)	−1.07 (1.21)	−1.42 (1.30)	−1.92 (1.62)	−1.26 (1.39)
Mean (SD) triglycerides, mmol/l	−0.03 (0.91)	−0.11 (1.45)	−0.24 (1.18)	−0.58 (1.62)	−0.24 (1.33)
Mean (SD) albumin:creatinine ratio	1.08 (6.87)	1.79 (17.38)	0.16 (24.86)	2.95 (29.53)	1.49 (21.30)
% Change in proportion prescribed glucose-lowering drug	53	56	63	76	61
% Change in proportion prescribed BP-lowering drug	25	32	35	43	34
% Change in proportion prescribed lipid-lowering drug	62	63	69	65	64

**Change adjusted for age, sex, ethnicity, randomisation group, and low education, (95% CIs)**					
BMI in kg/m^2^	−0.2 (−0.4 to 0.05)	−0.7 (−0.9 to −0.5)	−0.7 (−0.1 to −0.5)	−0.1 (−0.5 to 0.2)	−0.5 (−0.6 to −0.4)
Mean (SD) systolic BP, mmHg	−3.5 (−5.7 to −1.3)	−8.7 (−10.5 to −7.0)	−14.8 (−16.9 to −12.8)	−20.5 (−23.9 to −17.0)	−12.0 (−13.1 to −10.8)
Mean HbA1c, %	0.1 (0.05 to 0.2)	−0.1 (−0.2 to 0.01)	−0.6 (−0.8 to −0.5)	−1.5 (−1.7 to −1.2)	−0.4 (−0.44 to −0.3)
Mean (SD) total cholesterol:HDL ratio	−0.5 (−0.7 to −0.4)	−1.1 (−1.2 to −1.0)	−1.5 (−1.6 to −1.4)	−2.3 (−2.5 to −2.2)	−1.3 (−1.4 to −1.2)
Mean triglycerides in mmol/l	0.04 (−0.05 to 0.1)	−0.1 (−0.2 to 0.04)	−0.2 (−0.4 to −0.1)	−0.6 (−0.7 to −0.4)	−0.2 (−0.3 to −0.2)
Mean albumin:creatinine ratio	1.3 (0.7 to 2.0)	0.5 (0.2 to 0.9)	0.0 (−1.6 to 1.5)	1.0 (0.1 to 1.9)	1.0 (0.3 to 1.8)

**Predicted probability of being prescribed medication at 5 years (if not prescribed at baseline)^a^ (95% CIs)**					
Prescribed any glucose-lowering drug	0.38 (0.31 to 0.44)	0.54 (0.50 to 0.59)	0.69 (0.64 to 0.74)	0.86 (0.81 to 0.90)	0.62 (0.60 to 0.65)
Prescribed any BP-lowering drug	0.21 (0.16 to 0.25)	0.28 (0.24 to 0.33)	0.42 (0.36 to 0.48)	0.50 (0.44 to 57)	0.36 (0.33 to 0.39)
Prescribed any lipid-lowering drug	0.55 (0.48 to 0.62)	0.68 (0.63 to 0.73)	0.76 (0.70 to 0.81)	0.71 (0.65 to 0.78)	0.69 (0.66 to 0.71)

a Adjusted for age, sex, ethnicity, randomisation group, and age of leaving full-time education, BMI = body mass index. BP = blood pressure. CVD = cardiovascular disease. HbA1c = glycated haemoglobin. HDL = high-density lipoprotein. SD = standard deviation.

### Glycated haemoglobin (HbA1c)

Baseline median HbA1c ranged from 6.2% in Q1 to 7.2% in Q4 (Table 2). A significant increase in HbA1c was observed in Q1 (+0.1%; 95% CI = 0.05 to 0.2) over 5 years of follow-up (Table 3). There was no change in HbA1c levels in Q2, while large reductions were seen in Q3 (−0.6%; 95% CI = −0.8% to −0.5%) and Q4 (−1.5%; 95% CI = −1.7% to −1.2%) (Table 3).

### Systolic blood pressure

Baseline systolic blood pressure ranged from 137 mmHg (SD = 17) in Q1 to 161 mmHg (SD = 24 mmHg) in Q4 (Table 2). Over 5 years follow-up the smallest reduction was observed in Q1 (−3.5 mmHg; 95% CI = −5.7 mmHg to −1.3 mmHg) and the largest reduction in Q4 (−20.5 mmHg; 95% CI = −23.9 mmHg to −17.0 mmHg) (Table 3).

### Total:HDL (high-density lipoprotein) cholesterol ratio

The mean (SD) total:HDL cholesterol ratio was 3.8 (1.1) in Q1 at baseline and 5.7 (1.6) in Q4 (Table 2). From diagnosis to 5-year follow-up, the total:HDL cholesterol ratio decreased in all four quartiles, with the smallest reduction in Q1 (−0.5; 95% CI −0.7 to −0.4) and the largest in Q4 (−2.3; 95% CI = −2.5 to −2.2) (Table 3).

### Triglycerides

At diagnosis, median triglyceride levels ranged from 1.4 mmol/l in Q1 to 2.1 mmol/l in Q4 (Table 2). At 5 years, triglyceride levels had decreased in Q3 (−0.2 mmol/l; 95% CI = −0.4 mmol/l to −0. mmol/l) and Q4 (−0.6 mmol/l; 95% CI = −0.7 mmol/l to −0.4 mmol/l), with no change observed in Q1 and Q2 (Table 3).

### Albumin:creatinine ratio

Median albumin:creatinine ratio at baseline ranged from 0.7 mg/mmol in Q1 to 1.4 mg/ mmol in Q4 (Table 2). At 5-year follow-up significant increases were observed in Q1 (+1.3 mg/mmol; 95% CI = 0.7 mg/mmol to 2.0 mg/mmol), Q2 (+0.5 mg/mmol; 95% CI = 0.2 mg/mmol to 0.9 mg/mmol), and Q4 (+1.0 mg/mmol; 95% CI = 0.1 mg/mmol to 1.9 mg/mmol). No change was noted in Q3 (Table 3).

### Predicted probability of being allocated pharmacotherapy

The predicted probability of being prescribed cardioprotective medication at 5 years was higher in all four quartiles (Table 3). Those at the highest baseline modelled CVD risk were most likely to be prescribed cardioprotective treatment at 5 years (Table 3).

### Socioeconomic patterning

No association between low education or employment status and change in CVD risk factors was present within any of the quartiles of baseline-modelled CVD risk.

### Intervention effect

A sensitivity analysis excluding practices that received the intervention (promotion of intensive multifactorial diabetes care) demonstrated a non-significant decrease in systolic blood pressure in Q1 (−2.9 mmHg; 95% CI = −6.2 mmHg to 0.5 mmHg), and an increase in triglycerides in Q1 (0.2 mmol/l; 95% CI = 0.04 mmol/l to 0.3 mmol/l). Results otherwise suggested that the treatment groups could be pooled.

## DISCUSSION

### Summary

There was large variation in modelled CVD risk at diagnosis among this group of individuals with screen-detected diabetes. Compared to those at lowest risk, individuals in the highest modelled CVD risk quartile were more likely to be older, male, and smokers and to have a low education status. There was no difference in the proportion of participants prescribed cardioprotective drugs across the CVD risk quartiles at baseline. The largest reductions in modelled risk were seen in participants who were in the highest quartile of CVD risk at baseline, suggesting that treatment was offered to those at highest risk. For lipid-, glucose-, and blood pressure-lowering medication, those at highest CVD risk at baseline were most likely to be prescribed cardioprotective therapy at 5 years. Participants in the lowest quartile of risk at baseline had very similar levels of modelled CVD risk at 5-year follow-up and showed the least variation in change in modelled risk. There was no variation in change in modelled CVD risk or prescription of cardioprotective treatment by socioeconomic status, suggesting that treatment was equitable.

### Strengths and limitations

Data were collected from a large, representative population-based sample in three different European countries. There was high participant retention and little difference between individuals with and without follow-up data. Centrally trained staff collected data according to standard operating procedures. Recruitment of practices to the study was by self-selection, which may limit the generalisability of the study findings, but the baseline characteristics of the sample were nationally representative.^9^ The study population was largely white, and so it was not possible to assess treatment inequity in relation to ethnicity. As only 48 CVD-related deaths occurred between diagnosis and 5 years, they probably introduced a minimal amount of bias. The UKPDS risk model is one of the most extensively validated risk scores for use in European populations with diabetes.^30^,^31^ While it has been shown to overestimate risk in some contemporary populations with diabetes,^31^ it is effective at ranking individuals (discrimination) and is therefore suitable for examination of characteristics by risk quartile and resource prioritisation.

Presenting the data by quartiles of baseline CVD risk could potentially lead to regression toward the mean.^29^ To explore this effect, the baseline value of each risk factor was plotted against the change at 5 years. The lack of reduction in change in the tails suggests that the change values in Q1 and Q4 were not falsely attenuated. Clinical measurements were collected in triplicate, which may have helped reduce the potential for regression to the mean. The change in each risk factor was normally distributed within each quartile, and sensitivity analyses suggested that the quartiles represented the underlying patterns in an easily interpretable manner.

### Comparison with existing literature

The adverse CVD risk profile at baseline in the ADDITION-Europe cohort has been observed in cohorts of individuals with newly-diagnosed diabetes.

After 5 years of follow-up in ADDITION-Europe, the largest reductions in modelled CVD risk were seen in participants who were in the highest quartile of risk at baseline. These findings support data from the UKPDS^32^ and the Swedish National Diabetes register,^33^ which suggest that the greatest improvements in cardiovascular risk factors were seen among individuals with the highest initial values after diagnosis of diabetes. In the UKPDS, after an initial very large reduction in HbA1c levels, HbA1c slowly increased over the first 6 years in both intervention arms,^34^ and a sub-cohort of overweight individuals,^35^ while a more gradual decline in systolic blood pressure values was observed in the 9 years after diagnosis.^4^ In the more recent DESMOND (Diabetes Education and Self-Management for Ongoing and Newly Diagnosed) study,^36^ in which baseline information was collected up to 6 weeks after diagnosis,^37^ a similar pattern of a reduction in HbA1c, followed by a gradual increase, was observed.^36^

After 5 years of follow-up, ADDITION participants at highest baseline risk were more likely to be prescribed lipid-, glucose- or blood pressure-lowering drugs, after adjusting for several demographic covariates, including age, that may influence pharmacotherapy decisions by practitioners.^38^ This is in line with the finding that those at highest risk at baseline in the Danish ADDITION cohort had near-normal all-cause mortality after 7 years of follow-up, while those at lower risk had an all-cause mortality that was approximately twice as high.^10^ While the overall proportion of participants receiving cardioprotective medication could have been higher, the findings of the present study suggest that the ADDITION intervention was effective at reducing social inequity in treatment

### Implications for research and practice

The findings of this study suggest that the calculation of modelled CVD risk is a useful tool for guiding treatment decisions in newly-diagnosed patients with diabetes. Identifying who is at highest risk will help target treatment to those who need it the most, and is likely to lead to a reduction in treatment inequity.^42^ The group identified at high risk in the study cohort had the highest prevalence of stroke and myocardial infarction at baseline and therefore had the greatest capacity to change. Intensive treatment by lifestyle intervention and prescription of cardioprotective medication is likely to lead to clinically important reductions in CVD risk factors and modelled CVD risk, particularly in individuals with a high CVD risk at diagnosis.

Among individuals with low CVD risk at diagnosis, an early-treatment approach is likely to offset the expected age and/ or diabetes duration-related increase in modelled CVD risk. However, there is some evidence from the ADDITION-Denmark cohort to suggest that individuals at low risk are not being treated appropriately, leading to higher all-cause mortality compared to that for those at higher risk.^10^ Calculation of modelled CVD risk can also aid individualised patient goal setting and empowerment of self-care.^18^,^38^ This analysis provides a reference point for patients, clinicians, and policymakers when considering goals for changes in risk factors early in the course of the disease that account for the diverse cardiometabolic profile present in newly-diagnosed patients. Further analysis characterising CVD risk-factor trajectories could aid in both refining realistic goals for patients and identifying patterns that would allow a more nuanced approach to CVD risk-prevention initiatives.

## References

[1] Diabetes UK (2012). Early identification of people with and at high risk of type 2 diabetes and interventions for those at high risk.

[2] NHS Health Check Programme (2009). Putting prevention first — NHS health check: vascular risk assessment and management best practice guidance.

[3] UK Prospective Diabetes Study Group (1998). Intensive blood-glucose control with sulphonylureas or insulin compared with conventional treatment and risk of complications in patients with type 2 diabetes (UKPDS 33). UK Prospective Diabetes Study (UKPDS) Group. Lancet.

[4] UK Prospective Diabetes Study Group (1998). Tight blood pressure control and risk of macrovascular and microvascular complications in type 2 diabetes: UKPDS 38. UK Prospective Diabetes Study Group. BMJ.

[5] Heart Outcomes Prevention Evaluation Study Investigators (2000). Effects of ramipril on cardiovascular and microvascular outcomes in people with diabetes mellitus: results of the HOPE study and MICRO-HOPE substudy. Heart Outcomes Prevention Evaluation Study Investigators. Lancet.

[6] Pyorala K, Pedersen TR, Kjekshus J (1997). Cholesterol lowering with simvastatin improves prognosis of diabetic patients with coronary heart disease. A subgroup analysis of the Scandinavian Simvastatin Survival Study (4S). Diabetes Care.

[7] Gaede P, Lund-Andersen H, Parving HH, Pedersen O (2008). Effect of a multifactorial intervention on mortality in type 2 diabetes. N Engl J Med.

[8] Charles M, Simmons RK, Williams KM (2012). Cardiovascular risk reduction following diagnosis of diabetes by screening: 1-year results from the ADDITION-Cambridge trial cohort. Br J Gen Pract.

[9] Griffin SJ, Borch-Johnsen K, Davies MJ (2011). Effect of early intensive multifactorial therapy on 5-year cardiovascular outcomes in individuals with type 2 diabetes detected by screening (ADDITION-Europe): a cluster-randomised trial. Lancet.

[10] Lauritzen T, Sandbaek A, Carlsen AH, Borch-Johnsen K (2012). All-cause mortality and pharmacological treatment intensity following a high risk screening program for diabetes. A 6.6 year follow-up of the ADDITION study, Denmark. Prim Care Diabetes.

[11] Murphy E, Kinmonth AL (1995). No symptoms, no problem? Patients’ understandings of non-insulin dependent diabetes. Fam Pract.

[12] Wienbergen H, Senges J, Gitt AK (2008). Should we prescribe statin and aspirin for every diabetic patient? Is it time for a polypill?. Diabetes Care.

[13] Espelt A, Borrell C, Roskam AJ (2008). Socioeconomic inequalities in diabetes mellitus across Europe at the beginning of the 21st century. Diabetologia.

[14] Lauritzen T, Griffin S, Borch-Johnsen K (2000). The ADDITION study: proposed trial of the cost-effectiveness of an intensive multifactorial intervention on morbidity and mortality among people with Type 2 diabetes detected by screening. Int J Obes Relat Metab Disord.

[15] Echouffo-Tcheugui JB, Simmons RK, Williams KM (2009). The ADDITION-Cambridge trial protocol: a cluster-randomised controlled trial of screening for type 2 diabetes and intensive treatment for screen-detected patients. BMC Public Health.

[16] Webb DR, Khunti K, Srinivasan B (2010). Rationale and design of the ADDITION-Leicester study, a systematic screening programme and randomised controlled trial of multi-factorial cardiovascular risk intervention in people with type 2 diabetes mellitus detected by screening. Trials.

[17] Janssen PG, Gorter KJ, Stolk RP, Rutten GE (2009). Randomised controlled trial of intensive multifactorial treatment for cardiovascular risk in patients with screen-detected type 2 diabetes: 1-year data from the ADDITION Netherlands study. Br J Gen Pract.

[18] National Institute for Health and Clinical Excellence (2008). Type 2 diabetes: national clinical guideline for management in primary and secondary care (update).

[19] Royal College of General Practitioners in Denmark (1999). Type 2-diabetes in general practice — diagnosis and treatment.

[20] McIntosh AH, Home P, Brown F (2001). Clinical guidelines and evidence review for type 2 diabetes: management of blood glucose.

[21] Rutten G, Verhoeven S, Heine R (1999). NHG standards for treating type 2 diabetes (first revision). Huisarts Wet.

[22] Perk J, De Backer G, Gohlke H (2012). European guidelines on cardiovascular disease prevention in clinical practice (version 2012): the fifth joint task force of the European society of cardiology and other societies on cardiovascular disease prevention in clinical practice (constituted by representatives of nine societies and by invited experts). Int J Behav Med.

[23] Khunti K, Gadsby R, Millett C (2007). Quality of diabetes care in the UK: comparison of published quality-of-care reports with results of the Quality and Outcomes Framework for Diabetes. Diabet Med.

[24] Drivsholm T, Snorgaard O (2012). Organisation of treatment and control of type 2 diabetic patients. Ugeskr Laeger.

[25] Collins R, Armitage J, Parish S (2004). Effects of cholesterol-lowering with simvastatin on stroke and other major vascular events in 20536 people with cerebrovascular disease or other high-risk conditions. Lancet.

[26] Gaede P, Vedel P, Parving HH, Pedersen O (1999). Intensified multifactorial intervention in patients with type 2 diabetes mellitus and microalbuminuria: the Steno type 2 randomised study. Lancet.

[27] Coleman R, Stevens R, Holman R (2012). Updated UKPDS risk engine that estimates primary and secondary cardiovascular disease risk in people with recently-diagnosed or established type 2 diabetes. Diabetes.

[28] Dolan P (1997). Modeling valuations for EuroQol health states. Med Care.

[29] Tu Y-K, Gilthorpe MS (2007). Revisiting the relation between change and initial value: a review and evaluation. Stat Med.

[30] Chamnan P, Simmons RK, Sharp SJ (2009). Cardiovascular risk assessment scores for people with diabetes: a systematic review. Diabetologia.

[31] van Dieren S, Peelen LM, Nothlings U (2011). External validation of the UK Prospective Diabetes Study (UKPDS) risk engine in patients with type 2 diabetes. Diabetologia.

[32] Clarke PM, Gray AM, Briggs A (2004). A model to estimate the lifetime health outcomes of patients with type 2 diabetes: the United Kingdom Prospective Diabetes Study (UKPDS) outcomes model (UKPDS no. 68). Diabetologia.

[33] Ahmad Kiadaliri A, Clarke PM, Gerdtham UG (2013). Predicting changes in cardiovascular risk factors in type 2 diabetes in the post-UKPDS era: longitudinal analysis of the Swedish National Diabetes Register. J Diabetes Res.

[34] Davis TME, Cull CA, Holman RR (2001). Relationship between ethnicity and glycemic control, lipid profiles, and blood pressure during the first 9 years of type 2 diabetes: UK Prospective Diabetes Study (UKPDS 55). Diabetes Care.

[35] Holman RR (2006). Long-term efficacy of sulfonylureas: a United Kingdom Prospective Diabetes Study perspective. Metabolism.

[36] Davies M, Heller S, Skinner T (2008). Effectiveness of the diabetes education and self management for ongoing and newly diagnosed (DESMOND) programme for people with newly diagnosed type 2 diabetes: cluster randomised controlled trial. BMJ.

[37] Khunti K, Gray LJ, Skinner T (2012). Effectiveness of a diabetes education and self management programme (DESMOND) for people with newly diagnosed type 2 diabetes mellitus: three year follow-up of a cluster randomised controlled trial in primary care. BMJ.

[38] American Diabetes Association (2013). Standards of medical care in diabetes – 2013. Diabetes Care.

[39] Winkley K, Thomas SM, Sivaprasad S (2013). The clinical characteristics at diagnosis of type 2 diabetes in a multi-ethnic population: the South London Diabetes cohort (SOUL-D). Diabetologia.

[40] Chaturvedi N, Jarrett J, Shipley MJ, Fuller JH (1998). Socioeconomic gradient in morbidity and mortality in people with diabetes: cohort study findings from the Whitehall study and the WHO multinational study of vascular disease in diabetes. BMJ.

[41] Zeh P, Sandhu HK, Cannaby AM, Sturt JA (2012). The impact of culturally competent diabetes care interventions for improving diabetes-related outcomes in ethnic minority groups: a systematic review. Diabet Med.

[42] British Cardiac Society, British Hypertension Society, Diabetes UK (2005). JBS 2: Joint British Societies’ guidelines on prevention of cardiovascular disease in clinical practice. Heart.

